# Perovskite Topological Lasers: A Brand New Combination

**DOI:** 10.3390/nano14010028

**Published:** 2023-12-21

**Authors:** Liangshen Wang, Lijie Wu, Yong Pan

**Affiliations:** College of Science, Xi’an University of Architecture & Technology, Xi’an 710055, China; myoptics@xauat.edu.cn (L.W.); wulijieli@xauat.edu.cn (L.W.)

**Keywords:** photonic chip, nanolaser, topological laser, perovskite

## Abstract

Nanolasers are the essential components of modern photonic chips due to their low power consumption, high energy efficiency and fast modulation. As nanotechnology has advanced, researchers have proposed a number of nanolasers operating at both wavelength and sub-wavelength scales for application as light sources in photonic chips. Despite the advances in chip technology, the quality of the optical cavity, the operating threshold and the mode of operation of the light source still limit its advanced development. Ensuring high-performance laser operation has become a challenge as device size has been significantly reduced. A potential solution to this problem is the emergence of a novel optical confinement mechanism using photonic topological insulator lasers. In addition, gain media materials with perovskite-like properties have shown great potential for lasers, a role that many other gain materials cannot fulfil. When combined with topological laser modes, perovskite materials offer new possibilities for the operation and emission mechanism of nanolasers. This study introduces the operating mechanism of topological lasers and the optical properties of perovskite materials. It then outlines the key features of their combination and discusses the principles, structures, applications and prospects of perovskite topological lasers, including the scientific hurdles they face. Finally, the future development of low-dimensional perovskite topological lasers is explored.

## 1. Introduction

Recently, there has been an increasing worldwide curiosity in cutting-edge science and technology, encompassing advancements such as photonic chips and quantum computing. The swift advancement of photonic chip technology is vital in a range of fields, ranging from national defence science and technology to industrial engineering, medical, healthcare and communication transmission [1]. To tackle the current scientific challenges presented by photonic chips, it is essential to prioritise the research on their fundamental component: the low-dimensional laser light source [2]. Unlike traditional lasers, low-dimensional lasers are micro- and nanoscale in size. They can be divided into the following two main categories: In the first category, a specific material acts as both a gain medium and a resonant cavity. When the material is excited, it can be directly oscillated and amplified [3]. The second category involves two different materials acting as the gain medium and the resonant cavity and operates on the same emission principle as macroscopic lasers [4]. Recent advances in integrated photonic/quantum chips require low-dimensional light sources to achieve a threshold below kW/cm^2^ [5,6]. Smaller sizes are preferred if the output quality is assured. Based on reference [6], reduced threshold and improved stability in a light source can result in reduced power consumption, faster signalling, faster chip operation and improved parallel computing capability and transmission efficiency. However, the advances in chip technology are still limited by various factors like the quality of the optical cavity in the light source and the operating threshold and mode. The absence of a novel excitation method that fulfils the laser operational requirements for low energy consumption whilst remarkably reducing the device’s size is deemed a major hindrance, as reported in [7].

The development of topological optics has led to innovative ideas for new excitation mechanisms for low-dimensional lasers [8]. Topological lasers emit coherent light using their non-trivial energy band topology. This makes them robust to disorders and defects [9]. This topological protection significantly improves laser performance and allows for single-mode lasers with high robustness [10]. For example, topological photonic crystals can achieve efficient utilization of light energy by modifying the wave-particle duality of photons. This could solve the above-mentioned bottleneck [11]. Currently, research on topological nanophononics is not only addressing the issues of low-dimensional laser size and performance but also paving the way for large-scale integrated optical chip light sources [12]. In general, halide perovskites have the formula ABX_3_, where A is a monovalent cation, such as formamidine (FA^+^), methylammonium (MA^+^), caesium (Cs^+^) or rubidium (Rb^+^), and B is a divalent metal cation. Such as tin (Sn^2+^), bismuth (Bi^3+^) or lead (Pb^2+^), and X is a halogen anion, such as chlorine (CI^−^), bromine (Br^−^), iodine (I^−^) or mixtures thereof. Depending on the composition, the usually perovskites can be divided into the following two categories: organic–inorganic hybrid perovskites and pure inorganic perovskites. These materials have attracted attention in the context of gain media due to their distinctive laser gain properties, including high quantum yield, wide bandgap tunability and easy absorption properties [13]. Since 2019, various types of perovskite materials have demonstrated significant potential as laser-active region dielectric materials. Compared to many other active materials, the solution-treated films of these materials have the advantages of large optical absorption cross-section, large exciton binding energy, high photoluminescence quantum yield, high defect tolerance, and device tunability, making their role as a laser gain medium unparalleled [14]. As a result, the combination of topological optics and perovskite is set to lead to new breakthroughs in the research of micro- and nanolasers [15]. Thus, summarizing the topological lasers of low-dimensional perovskites is crucial.

**Figure 1:**
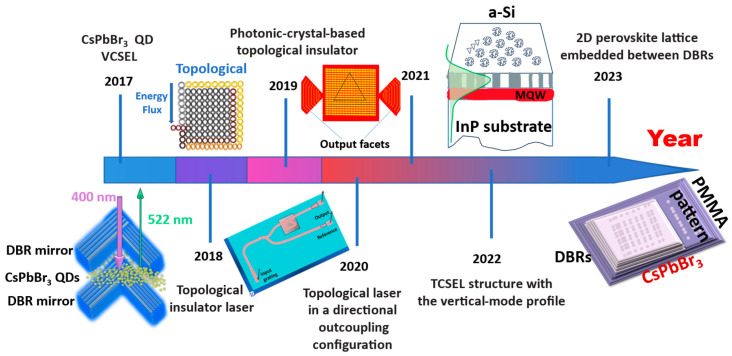
Recent process summarizes in topological lasers. Schematic of the CsPbBr_3_ Perovskite Quantum Dot Vertical Cavity Lasers, reproduced with permission from [7], copyright 2017, *ACS Photonics*. Laser mode in a topological cavity, reproduced with permission from [8], copyright 2018, *Science*. Schematic of the photonic crystal-based topological insulator, reproduced with permission from [9], copyright 2019, *Nature Nanotechnology*. Topological laser in a directional outcoupling configuration, reproduced with permission from [10], copyright 2020 *Nature*. Topological-cavity surface-emitting laser structure with the vertical-mode profile in green, reproduced with permission from [11], copyright 2022, *Nature photonics*. Illustration of the 2D perovskite lattice embedded between distributed Bragg reflectors (DBRs), reproduced with permission from [12], copyright 2023, *Science Advances*.

This study focuses on low-dimensional lasers using perovskite material as the primary gain medium and the topological mode as the excitation. Recent advances in perovskite topological lasers are summarised in terms of their principle, structure, application and prospects. This paper aims to introduce the coupling principle of perovskite materials and topological laser modes. It does so by summarising the current research on perovskite topological lasers and, accordingly, by expanding the depth of research at the photonic quantum scale. The study provides some ideas for the advancement of research on perovskite topological lasers. Figure 1 shows the milestones in the development of topological lasers in recent years [7,8,9,10,11,12].

## 2. Fundamental Principles of Topological Lasers

A laser is a coherent light source with exceptional luminous intensity and perfect directionality. A basic laser system comprises a pump source, gain medium and resonator. This three-part system is widely recognized within the field. In this systematic arrangement, the pump source provides the light source for the laser, while the gain medium absorbs the energy provided by the pump source and enhances the light. The resonator functions as the circuit between the pump source and the gain medium, and the output laser is chosen by oscillating the cavity [16]. Topology pertains to the examination of the characteristics of geometric figures or spaces that can retain their structure after being subjected to continuous shape changes. It focuses solely on the spatial relationships between objects, regardless of their shape and size [17]. As the notion of topology broadened from condensed matter physics to photon physics, a new genre of laser, known as the topological laser, emerged. The laser was inspired by the discovery of a topological insulator, in which interface electrons do not dissipate even in the presence of impurities. Similarly, a well-designed wave vector space topology can be used to create optical transport interfaces that support useful and interesting properties. In particular, this suggests that unidirectional waveguides allow light to flow around large defects without back reflection. Other types of topological lasers are also used for display purposes, the light can efficiently be directed around corners and defects, moving in a constant direction at the optical waveguide’s edge [18].

To date, the comparison system categorises topological lasers into the following three distinct categories: topological insulator lasers, topological defect lasers and radiation topological lasers. The first category can be further subdivided into quantum Hall lasers, quantum spin Hall lasers, quantum valley Hall lasers and lasers based on Su–Schrieffer–Heeger (SSH)-like models. The topological insulator laser uses a topology-protected edge mode laser to effectively bypass defects and corners. Among them, the topological edge state appears in the spatial boundary between the systems with different topologies, and the energy of the edge state is located in the gap of the bulk bands [19,20]. The continuous system of a laser with topological defects is quenched to an ordered state by competing time scales during the phase transition. These defects limit the consistency of the system and its ability to approach a fully ordered state, the free spectral range is larger than the conventional micro-/nanowire cavity, so it is more likely to support single-mode laser emissions in a specific spectral gain window [21]. The radial and azimuthal polarization vector beams can be directly emitted by the radiation topological laser with square lattice photonic crystals to manipulate the polarization and angular momentum of the light with non-trivial topological charges [22,23]. Quantum Hall lasers combine two photonic crystals with different topologies, one with a non-trivial topology activated by an external magnetic field and the other with trivial topology, the photon Quantum Hall effect dictates that a topologically protected edge mode must exist along the interface [19,24]. Photons in a photonic system can exhibit spin-like quantities, called pseudo-spins, which are represented by two internal states (e.g., two polarised states or two orbital modes in a cavity). Spin-momentum locked edge mode lasers are typical of quantum spin Hall lasers. These devices consist of two cellular lattice photonic crystals (PhC), in which the inner and outer PhC regions have the same bandgap, but the topology is different [25]. The room temperature laser from the photonic Valley Hall nanocavity, based on the quantum Valley Hall effect, has a narrow spectrum, high coherence and threshold behaviour. The emitted beam has a singularity encoded by the three-terminal cavity mode, which exists in the bandgap of the two Houle periodic photonic lattices with opposite parity disorder [26]. The orbital version of the SSHHamiltonian is realised in a one-dimensional polaron microcolumnar lattice, where laser emissions from the topological edge state persist despite local lattice deformation. Under non-resonant optical pumping, the gain occurs in the topological edge state. The topological robustness of the laser interaction to the optically induced lattice deformation is then demonstrated using the polariton interaction [27]. Irrespective of the laser’s topology, the resonator’s structure has been enhanced to attain more efficient and stable optical resonance and beam quality. Selecting the appropriate gain medium is vital to the laser’s performance and output characteristics. 

Generally, gain media must fulfil basic requirements such as high triplet level, low excitation threshold, good spectral matching and effective population inversion [11]. Perovskites can be classified into the following four types based on their dimensionality: 0-dimensional quantum dots, 1-dimensional structures, 2-dimensional microstructures and 3-dimensional structures. The quantum dots that won this year’s Nobel Prize in Chemistry are small but powerful semiconductor particles with exceptional optical and electronic properties, typically less than 20 nanometres in size. To put this in perspective, they are 110,000 times smaller than the diameter of an average human hair. Because of their tiny size, quantum mechanics comes into play when measuring their properties. The 0D perovskite quantum dots (QDs) have found wide application in lasers due to their remarkable properties, including convenient synthesis, high luminescence quantum yield, robust tolerance to defects and tunable bandgap of different elements [28]. One-dimensional (1D) perovskite, when used as the gain medium in a topology laser, boasts various advantageous characteristics, including the ability for a micro-laser with an ultra-thin gain medium (<50 nm) to produce single-mode emission with a low laser threshold (6.8 μJ/cm^2^). In addition, laser emission can be continuously tuned in the gain spectrum range (from λ = 532 to 519 nm) by altering the thickness of the gain medium. This innovation addresses common issues found in thick quantum dot films, such as poor uniformity, aggregation and luminescence quenching [29]. In the two-dimensional structure of perovskite, the nanostructures—known as nanoplates (NPL)—sustain quantum confinement in a singular direction. The electronic properties of these materials are characterised by narrow emission lines at low and room temperatures, with significantly improved exciton binding energy and absorption cross sections (corresponding to the area of NPL) compared to colloidal quantum dots. Additionally, the low threshold for amplified emission and optical gain coefficient provided by simultaneous one-photon and two-photon absorption pumping is approximately four times greater than that offered by the colloidal quantum dots [30]. Although 3D perovskite possesses a high optical gain and a low threshold, it can only achieve high-efficiency laser emission due to its narrow band gap and poor thermal conductivity. This hinders its ability to achieve high-energy laser emission and adversely affects its performance and operational life [31]. After careful consideration and thorough comparison, our recommendation is to utilize a sub-2D low-dimensional perovskite as the gain medium.

The reason why the combination of low-dimensional perovskites and topological lasers can be improved is mainly due to the special properties of low-dimensional perovskites and the unique properties of topological lasers. First, low-dimensional perovskites have smaller dimensions, usually only a few nanometres to a few hundred nanometres, and therefore have a higher specific surface area and quantum confinement effects. These properties make low-dimensional perovskites have a higher optical gain and a lower threshold, and can realise high-efficiency laser emissions [32]. Secondly, the topological laser uses the edge states in the topological material to realize the unidirectional transmission of light. It has a strong robustness and can keep stable performance under the design of different geometric resonators [33]. The combination of low-dimensional perovskite and topological laser can make full use of the advantages of both to achieve high efficiency, high power, low threshold, stable and tunable laser emission, expanding the application of topological laser.

Table 1 summarizes the performance comparison of nanolasers with different working materials reported in recent years. Nanolasers are similar to the gain medium used in zero topology lasers and have some reference value. It is not difficult to see from Table 1 that nanolasers with perovskite gain medium do not have a high-quality factor, but they do have a visible emission wavelength, which is of great importance for future applications. The topology of the laser is very conducive to the optical emission of the perovskite (structure) material. On the one hand, perovskite (structure) quantum dots can effectively absorb light energy and generate excitons due to their excellent optical and chemical properties, large light absorption cross-sections and exciton binding energy. On the other hand, the wavelength of the quantum dots can be tuned to the blue spectral region, resulting in a broad spectrum of light emission. Topological lasers rely on structural robustness to achieve high-efficiency beam delivery. As an example, topological photonic crystals are composed of a series of hexagonal rings. Each ring is coated with a layer of material doped with fluorescent substances, forming a miniature resonant cavity [34]. When light hits the topological photonic crystal, the vibrational modes and interaction laws of the fluorescent substance differ from those of ordinary light. This leads to a reduction in the probability of reflecting, scattering or absorbing a photon, resulting in q highly efficient beam transmission [35]. For clarity, consider a magneto-optical crystal consisting of a square lattice of yttrium–iron–garnet (YIG) rods with a radius of 0.11α in air, where α is the lattice constant. An external direct-current magnetic field applied in the out-of-plane (z) direction induces strong gyromagnetic anisotropy, with the permeability tensor taking the form (1):

(1) $$u=\left[\begin{array}{ccc} u & ik & 0\\ -ik & u & 0\\ 0 & 0 & u_{0} \end{array}\right]$$

In the above formula, k and u represent the permeability tensor, i is the imaginary unit, and $u_{0}$ is the permeability in vacuum. With a 1600 Gauss applied field, the tensor elements in YIG at 4.28 GHz are k = 12.4$u_{0}$ and u = 14$u_{0}$. This formula ignores the effects of material dispersion and loss and assumes that there are frequency-independent permeability tensors with real k and u values. Due to the existence of magnetic anisotropy, we have to apply the following traditional band theory of photonic crystals to the system: we remove the magnetic field from the Maxwell’s equations to obtain the following master Equation (2):

(2) $$\nabla \times (u^{-1}(r)\nabla \times E)=\varepsilon (r)\omega^{2}E$$

Here, the inverse permeability tensor $u^{-1}(r)$ and the scalar permittivity ε(r) are both functions of position, and ω is the mode frequency, and E is electric-field strength [36,37].

**Table 1:** Comparison of different gain media in a nanolasers.

Gain Medium	Threshold	Q-Factors	Output Wavelength (nm)	Published Year	Refs.
Oregon Green 488	0.005 mJ	14.8	531	2009	[38]
InGaN@GaN	3.7 kW/cm^2^	~17	510	2012	[39]
CH_3_NH_3_PbBr_3_	59 uJ/cm^2^	~855	~552	2016	[40]
PMMA:DCM	5.6 MW/cm^2^	310	~643	2017	[41]
NaYF^4^:Yb^3+^/Er^3+^	70 W/cm^2^	>200	664	2019	[42]
InAsP/InP	~12.5 kW/cm^2^	~35,000	~1550	2019	[43]
Ga_0.05_Al_0.95_As/Ga_0.8_Al_0.2_As	200 μW	~72,000	754	2021	[27]
CsPbBr_3_	8.95 μJ/cm^2^	~5000	515	2023	[17]

The transverse magnetic mode in a rotating magnetic photonic crystal can be formally mapped to the electron wave function in a periodic electromagnetic field, so the only requirement for the existence of a unidirectional edge mode is that the Chern number in all frequency bands below the gap is non-zero. In a two-dimensional system, the first Chern number characterizes the topological invariant, which is proportional to the Berry phase that encloses the first Brillouin zone. We can express the integral form of the topological Chern number in Equation (3).

(3) $$C_{n}=\frac{1}{2\pi }\int_{BZ}d^{2}k\Omega_{n}(k_{x},k_{y})$$

Here, $C_{n}$ is the Chern number, π is the Pi, $\Omega_{n}(k_{x},k_{y})$ is the Berry curvature, $k_{x}$ and $k_{y}$ are the crystal momentum k varying in the x and y directions, respectively, in the first Brillouin zone. According to the description of an integral quantum Hall state in band theory, it occurs when an electron confined to two dimensions is placed in a strong magnetic field. The quantization of an electron’s circular orbit with a cyclotron frequency $\omega_{c}$ leads to a quantized Landau energy level of $\varepsilon_{m}=\hbar \omega_{c}(m+1/2)$. If *N* Landau levels are filled and the remaining levels are empty, the gap will be separated from the occupied state as in an insulator. Unlike insulators, however, electric fields can cause cyclotron orbital drift, producing Hall currents characterized by quantifying Hall conductivity. Therein, Hall conductance is a physical quantity that measures the rate of change in current produced by the Hall electrode. It is an important parameter of the Hall effect and can be used to measure magnetic field, conductivity and other physical quantities. Its expression can be found in Equation (4) [44,45].

(4) $$\sigma_{xy}=-\frac{e^{2}}{h}\sum_{n}C_{n}$$

Here, $\sigma_{xy}$ is the hall conductance of two-dimensional insulators, e is basic charge, h is the Planck’s constant. The quantization of $\sigma_{xy}$ has been measured to 1 part in 10^9^. This precision is a manifestation of the topological nature of $\sigma_{xy}$ [46].

**Figure 2:**
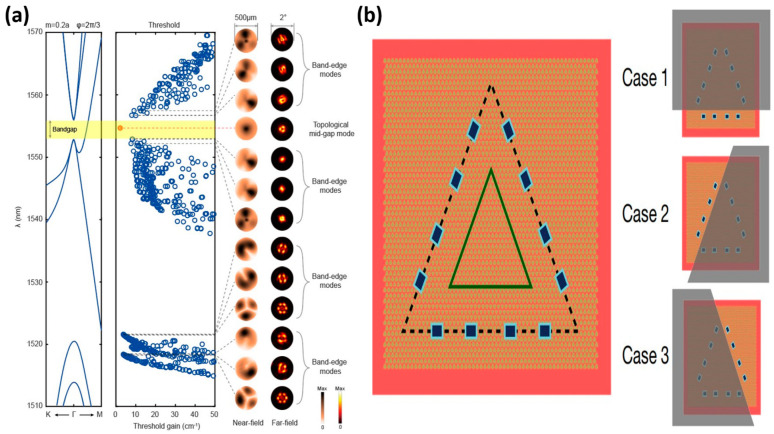
(**a**) Pattern analysis of TCSEL, reproduced with permission from [11], copyright 2022, *Nature photonics*. (**b**) A THz quantum cascade laser with topologically protected valley edge states, reproduced with permission from [10], copyright 2020, *Nature*.

Topological lasers are lasers that function using topologically protected edge-state currents. These edge-state currents travel in semicircular trajectories along the edges of topological insulators and are not susceptible to interruptions from impurities or defects [47]. Photons do not have magnetic moments and are, therefore, not directly affected by magnetic fields. However, a similar effect can be achieved by electrons excited by incident light. The magnetic field affects these electrons differently, so the light is also affected differently [48]. The principle of topological lasers is closely related to their pumping method, as mentioned in reference [14]. To pump a topological laser, energy must be injected into the topological insulator to excite the electrons and initiate the topologically protected edge state currents. The pumping method can be either electrical or optical. In electrical pumping, the electrons in the gain medium material are excited by incident light, which creates a jump and is then stimulated to emit radiation (as stated in reference [49]). The topological cavity surface-emitting laser (TCSEL) is a low-dimensional device that generates nanolasers by optical pumping, combining the structure of vertical surface-emitting lasers with the topological laser mode, as stated in reference [50]. To generate a stable single-mode laser over a larger area of the chip, the authors and their team successfully tackled this challenge by implementing a Paul Dirac vortex topology cavity. To be specific, to achieve far-field size emission above 500 μm with narrow scattering angles and to lower the excitation threshold for optical pumping (Figure 2a) [11], the effective size of the device is continuously increased by adding more photonic crystal cycles to its structure. In contrast to electric pumping that injects electrons and holes to make the gain medium absorb energy, the electrons of the gain medium of an electrically pumped terahertz are excited by applying voltage across the topological insulator [51]. Zeng et al. at Nanyang Technological University, Singapore, and Chattopadhyay et al. at the University of Leeds, United Kingdom, reported a terahertz laser based on the boundary states of the topological valley in 2020, as shown in Figure 2b [10]. An investigation into a two-dimensional bandgap valley material led to the creation of a compact photonic crystal with valley degrees of freedom. By simply superimposing one side of the device, it can be seen that each laser mode emits almost the same intensity from both sides. Consequently, the left- and right-handed cavity modes are both of equal intensity and are protected by the topology. To achieve effective energy transfer and topology-protected initiation of edge-state currents, it is, therefore, essential to select the appropriate light source and material for each of the two pumping modes [52].

**Figure 3:**
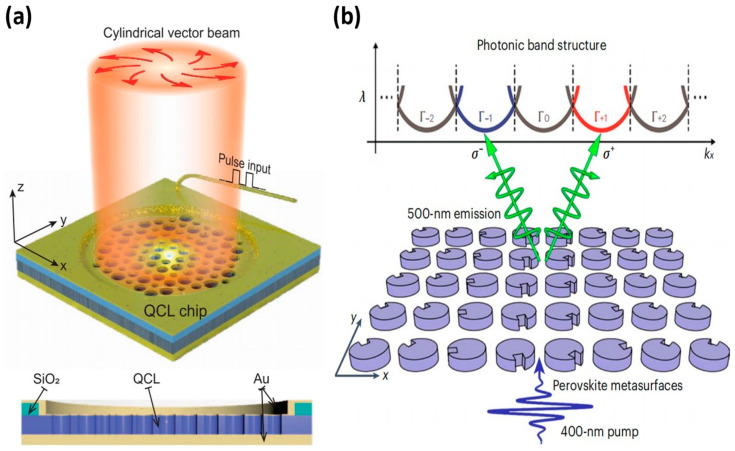
(**a**) Electro-pump quantum cascade laser (QCL) based on photonic Majorana zero-mode (MZM), reproduced with permission from [54], copyright 2023, *Nature Communications*. (**b**) Schematic representation of perovskite metasurfaces supporting spin-valley locking emission, reproduced with permission from [56], copyright 2023, *Nature Materials*.

Aside from conventional pump sources, chemical and nuclear excitation methods also exist. To enhance the coherent light produced by the pump source, a gain medium, as stated in reference [53], is indispensable. At present, silica and perovskite are the two main types of laser gain media that exhibit superior performance in producing topological laser modes. Figure 3a shows a topological cavity that carries a photonic MZM [54]. Three-well resonant phonon GaAs/Al_0.15_Ga_0.85_ terahertz QCL chip, such as the one used in this work, which is created by the passage of light through the top metal layer and hexagonal air-hole lattice of a terahertz QCL wafer. This structure effectively reduces intracavity reflection and loss, thereby improving the laser’s performance in terms of output and excitation. Due to the high refractive index property of silica, the laser output power and energy can be improved to some extent. However, the excessive refractive index of silica causes the light to be confined, resulting in insufficient oscillation capability, making it difficult to achieve low-threshold operation for topological lasers with silica as the gain medium in new photonic chips that require low energy consumption; therefore, a new material substitution is urgently needed [55].

In a topological laser, external excitation sources (such as light) excite the atoms or molecules in the perovskite material to high energy states. An ultrathin gain medium of all-inorganic caesium-lead-halide perovskite quantum dots is used to reverse the number of particle distributions, enabling laser amplification and emission [57]. The optical pumping mechanism of perovskite is influenced by its energy level structure, external factors such as temperature and pressure, and particle interactions. In addition, the topology of the perovskite can also influence the optical pumping mechanism. For example, a non-banal topology can improve the optical localisation and efficiency of optical pumping, ultimately leading to improved laser performance. The interface between perovskite and other materials affects the optical pumping mechanism, including charge and energy transfer. In addition, the spin-valley-locked perovskite mode has a topological structure that enables efficient and low-threshold broadband laser emission with tunable properties. Valley polarisation inversion is achieved by tuning the spin state, resulting in tunable nanolaser output. According to Figure 3b [56], the emission mechanism of spin valley-locked perovskite lasers is shown. The authors of the paper developed a self-limiting assembly method that allows for polycrystalline inclusion crystals to grow conformally within the hypersurface template. The conformal growth of the phenylethylammonium bromide (PEABr) inclusion ensures the conformal growth of inclusion crystals while limiting the grain size during the crystallization process. The characteristic size of the photonic structure can be as small as 50 nm, resulting in bright and uniform luminescence over a 40 × 40 μm^2^ area. This study demonstrates compact spin-valley-locked perovskite emissive surfaces that assign spin-dependent geometrical phases to bound states in the continuum via Brillouin zone folding and process photons with different spins selectively to opposite valleys. This topology of spin-valley-locked perovskites is believed to create possibilities for tunable laser emission. This approach can generate entangled photon pairs on-chip for use as a light source for topological lasers and is also expected to contribute to the realization of chiral light-emitting diodes [58].

**Figure 4:**
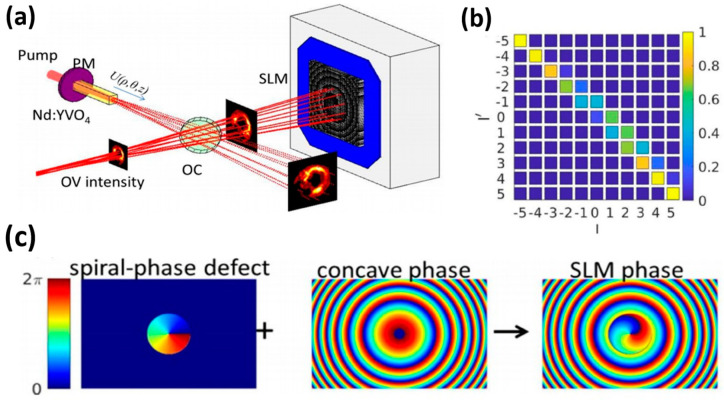
(**a**) Schematic representation of the laser formed by the pump mirror (PM) and the reflection type spatial light modulator (SLM). (**b**) Phase modulation provided by SLM and combining with limited helical and concave phases. (**c**) Phase modulation is provided by SLM and combines limited helical and concave phases, reproduced with permission from [59] copyright 2022, *IEEE Photonics Journal*.

A well-designed resonant cavity, in addition to the effect of the gain medium, is critical in increasing the gain factor of low-dimensional topological lasers. When designing resonant cavities, the parameters of the mode type, length, width and mode are some of the factors to be considered. Spiral resonant cavities, electric dipole moment resonant cavities, lattice resonant cavities and others are some examples of resonant cavities. The helical resonant cavity is a type of structure that uses optical field interaction to produce optical phenomena. This structure has a high tuning sensitivity and stability, making it ideal for topological laser modes. Lin et al. [59], from the National Sun Yat-sen University in Taiwan, conducted a study wherein they designed an optical resonator that uses a helical phase defect to generate vortex lasers, as illustrated in Figure 4a. Figure 4b shows that the spiral-phase-defect (SPD) has topological charges of −1 and +1. The sign and size of the topological charge can be chosen for the resonant vortex mode. In addition, the authors used theoretical calculations to obtain a parameter space for generating vortex beams with variable topological charges. They have also demonstrated that the optical resonator can produce stable vortex beams in a 1064 nm vortex laser system, as depicted in Figure 4c.

**Figure 5:**
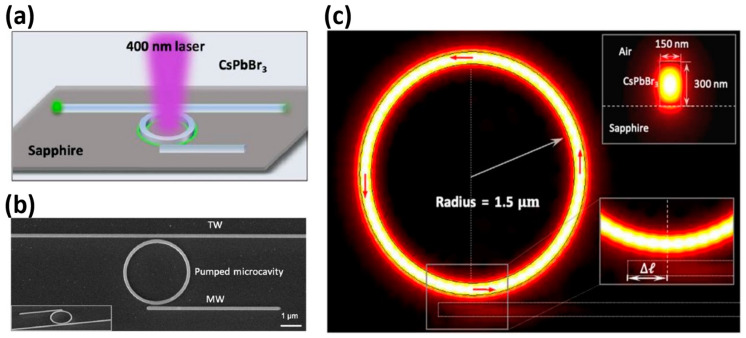
On-chip integrated visible light micro-nano laser, reproduced with permission from [60], copyright 2023, *Science Advances*: (**a**) Schematic of excitation of 400 nm optical pumped laser. (**b**) Top view and 52° tilt scanning electron microscope (SEM) images of CsPbBr_3_ microcavity coupling system (inset). (**c**) The electric field distribution of the intrinsic mode of a topological laser system is obtained by simulating the intrinsic frequency (the radius of the microcavity is 1.5 μm).

A scalable order strategy was reported by Liao et al. [60] from Tsinghua University to achieve a stable on-chip integrated visible micro-nano laser. The improvement of laser performance was realised through enhancing the structural order (n) of specialized surfaces, generating desirable topological patterns. Figure 5a displays a schematic outline of the on-chip integrated laser mechanism, in which a femtosecond pump light with a wavelength of 400 nm enters the ring for excitation. Figure 5b presents the SEM top-view image of the CsPbBr3 microcavity coupling system in the integrated laser mechanism. The electric field distribution in the intrinsic mode of the on-chip integrated laser system is shown in Figure 5c, illustrating a microcavity radius of 1.5 μm. The red arrow displays the time-averaged flow of power for the optical field in the microcavity, indicating strong unidirectional mode propagation. The lower right panel shows an enlarged view of the microcavity-MW coupling region. The above figure shows the simulated results of the modal dispersion of the optical field in the cross section of the CsPbBr_3_ waveguide (width: 150 nm; height: 300 nm), which are shown in the upper right section. The use of these resistors can lead to a significant improvement in the optical field quality factor and uniformity, which, according to the physical analysis, favours high-power and high-brightness laser applications. To achieve a higher optical field quality factor, it is necessary to optimise the resonator’s physical structure and material selection. In addition, the optical field uniformity can be improved by precisely controlling the resonator’s vibration modes. This study extends the research directions for non-Hermitian photonics in terms of nonlinear optics and topological photonics [61].

## 3. The Structure of Topological Lasers

**Figure 6:**
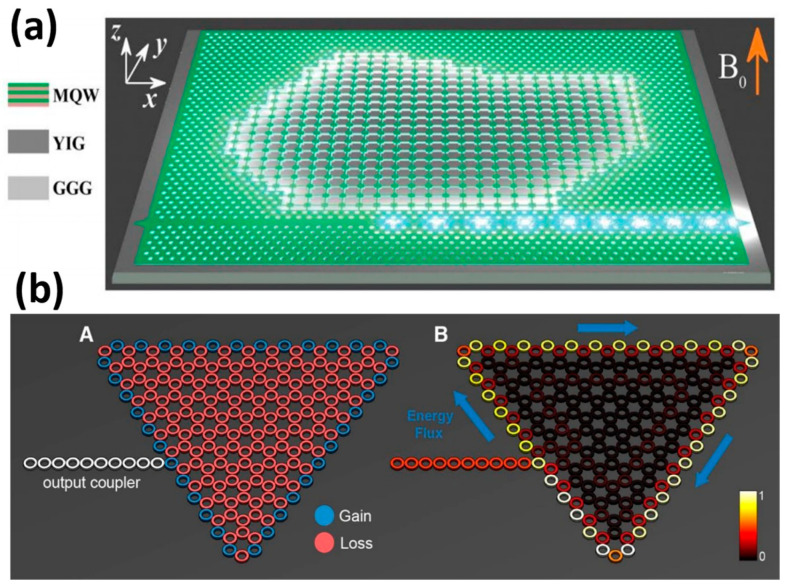
Schematic structure diagram of the topological laser: (**a**) A topological cavity of an arbitrary shape and an integration, reproduced with permission from [24], copyright 2017, *Science*. (**b**) Geometry and laser patterns of topological insulator lasers based on the Haldane model, (**A**) Cavity geometry (same for topological and trivial), (**B**) The steady state topological lasing mode of the topological cavity, reproduced with permission from [8], copyright 2018, *Science*.

In 2017, Bahari et al. [24] at the University of California, San Diego, CA, USA, demonstrated topological cavity lasers by combining geometrically independent and integrated nonreciprocal topological cavities (Figure 6a) with stimulated emissions from a unidirectional photonic edge state. The structures are made of InGaAsP multiple quantum wells (MQW), bonded on YIG grown on Gadolinium Gallium Garnett (GGG) by molecular beam epitaxy. An isolation ratio of over 10 dB was achieved at the output of a chosen waveguide. This experiment allows for the creation of intricate topological circuits with an arbitrary shape, which could greatly aid in the reliable and integrated transportation of photons in both classical and quantum regimes. In the subsequent year, the concept of topological insulator lasers was introduced by Harari et al. [8] (Figure 6b). Several types of topological lasers have been reported. This laser consists of several resonant rings. By adjusting the length of the resonant rings, an equivalent magnetic field is created that has opposite effects on clockwise and counterclockwise rotating light. Unlike the previous method, this laser breaks the symmetry of the time inversion of the photonic crystal [62], amplifying the pump light and eliminating the need for an additional static magnetic field, resulting in a double benefit. Moreover, by adding gains and losses to the structure, topologically protected lasing can be achieved without affecting the resulting topological properties, which is a theoretical breakthrough.

**Figure 7:**
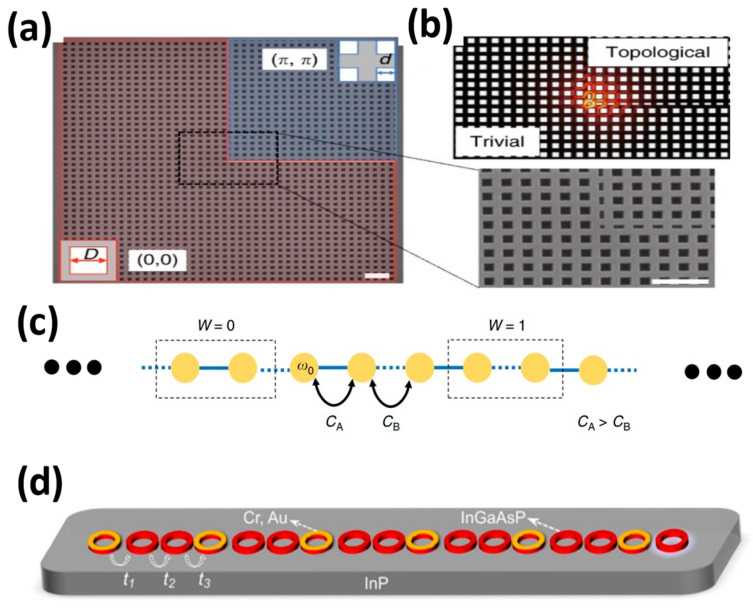
Structural diagram of the topological laser (The scale bar is 1µm): (**a**) SEM image of a square two-dimensional topological PhC cavity. (**b**) Topological angular electric field distribution, reproduced with permission from [63]. Copyright 2020, *Light:Science & Applications*. (**c**) One-dimensional SSH structure consisting of linear chains of dimeric unit cells, reproduced with permission from [42], copyright 2019, *Light:Science & Applications*. (**d**) A one-dimensional non-Hermitian photonic lattice composed of 17 InGaAsP microloops, reproduced with permission from [64], copyright 2023, *Physical Review Letters*.

Figure 7 displays the construction of a topological laser across various platforms. In their study, the authors designed a topological nanospace involving two types of PhC plates with square pores having the same period, but different unit cells, as depicted in Figure 7a [63]. While sharing a common band structure, the red and blue regions exhibit contrasting topologies, which are distinguished by the value of the two-dimensional Zak phase (*θ*_Zak_), determined from the integration of the Berry connection within the first Brillouin zone. By suitably tuning the gap distance between the trivial and nontrivial parts of the PhC slab, a higher Q factor can be achieved. In addition, Figure 7b depicts the electric field distribution of the corresponding angular state, showing significant confinement in the nanoscale and resulting in strong light-matter interactions. This characteristic has promising applications like developing topological nanolasers. Figure 7c illustrates a one-dimensional SSH structure [42]. The structure consists of linear chains of dimer unit cells comprising of identical resonators (with resonance frequency ω_0_) having staggered nearest-neighbour coupling strengths. The capacitances used are C_1_ = C_3_ = ----- = C_odd_ = C_A_ and C_2_ = C_4_ = ----- = C_even_ = C_B_ (with C_A_ being greater than C_B_). Figure 7d displays a one-dimensional non-Hermitian photonic lattice. It consists of 17 InGaAsP microrings (shown in red). Each microring consists of three microrings, exhibiting optical loss and gain, respectively [64]. The gain cavity comprises of an InGaAsP quantum well on an InP substrate, whereas the loss is realized by a layer of metal (Cr + Au) at the corresponding position.

**Figure 8:**
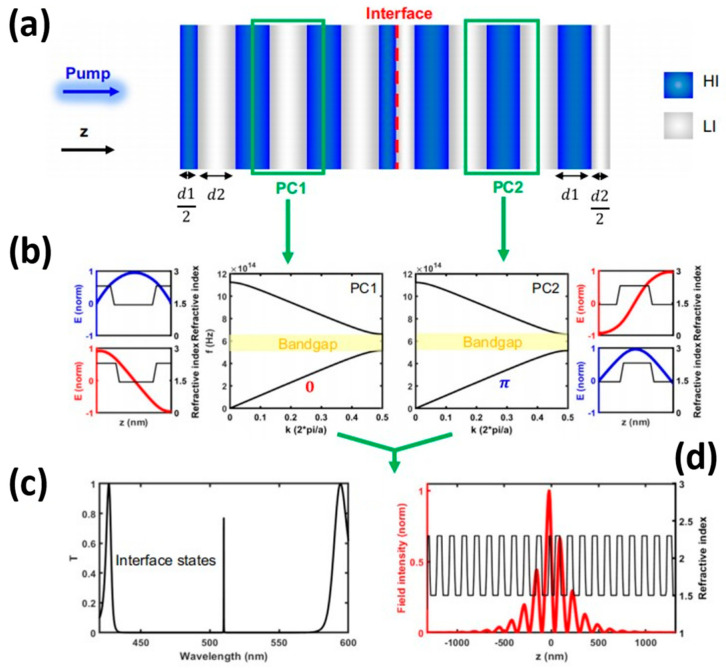
Design principle of a 1D topological microcavity, reproduced with permission from 17, copyright 2023, *Nature Communications*: (**a**) A 1D topological microcavity formed by two photonic crystals (PC1 and PC2) composed of high refractive index (HI) and low refractive index (LI) layers. (**b**) Normalized electric field distribution of the first two optical bands along the *z*-axis within the respective cell. (**c**)The high Q interface state appearing within the optical band gap; (**d**) Spatial distribution of the electric field in the microcavity.

In 2017, a group led by Wang [65] at Nanyang Technological University in Singapore successfully developed vertical-cavity surface-emitting lasers (VCSELs) utilizing CsPbX_3_ lead halide perovskite nanocrystals (IPNCs). These lasers are characterised by a low excitation threshold of 9 µJ/cm^2^, a directional output with a beam dispersion of approximately 3.6 degrees, and exhibit good stability. In the same year, Huang’s group [7] at the University of Washington in the United States reported an ultra-low lasing threshold of 0.39 µJ/cm^2^. The research team created a VCSELs laser incorporating CSB-bBr_3_ QDs in a thin film and two high-reflection distributed Bragg reflectors (DBRs). In 2023, Tian et al. from Nanyang Technological University in Singapore demonstrated a low-threshold single-mode laser emission with vertical emission. Their lithography-free, solution-processed and fully inorganic monolayer of lead halide perovskite quantum dots acted as an ultrathin gain medium. This experiment demonstrated the resilience of topological lasers to localised perturbations in multilayer structures. The cavity, as illustrated in Figure 8a, comprises an interface between two semi-infinite, one-dimensional binary photonic crystals, referred to as PC1 and PC2, where PC2 has inversion symmetry along the z-direction. The LI and HI present near the inversion centre display different Zak phases in the lowest optical band because of their varied positions (Figure 8b). The one-dimensional topological cavity consists of two 10-unit half-cavities positioned at the centre of the first optical bandgap. At approximately 515 nanometres, it exhibits a high-quality interface state with a Q-value above 5000. The transmission spectrum calculated is illustrated in Figure 8c, with the electric field heavily confined to the interface of the two photonic crystals (PCs). Moreover, the asymmetrical distribution reaches its peak within the first Hole-Injection (HI) layer of PC1, which is depicted in Figure 8d.

## 4. Applications and Prospects of Topological Lasers

Topological lasers are a novel optical device that offer several advantages, including high efficiency, power and beam quality [66]. As a result, topological lasers have found widespread application in all-optical switching, optical communication, TCSEL, Terahertz silicon interconnection technology, silicon-based photonic circuits and other areas.

**Figure 9:**
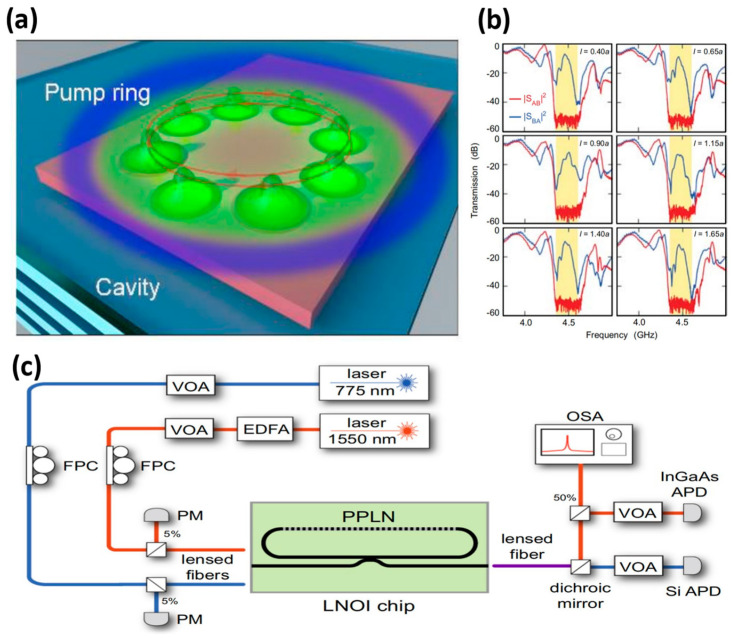
Application scenario of the topological laser: (**a**) A schematic representation of the formation of a petal-shaped condensed pattern, reproduced with permission from [67], copyright 2023, *ACS Photonic*. (**b**) Normalized electric field distribution of the first two optical bands along the *z*-axis within the respective cell, reproduced with permission from [68], copyright 2009, *Nature*. (**c**) Integrated of resonant second-order nonlinear optical devices, reproduced with permission from [69], copyright 2022, *Nature Communications*.

Topological lasers accomplish the amplification and phase modulation of coherent light by generating phase and amplitude disparities on the surface of the laser cavity, enabling all-optical switching. The resulting phase-modulated light can be harnessed in all-optical switches, facilitating the processing and transmission of optical signals. Zhang et al. [67] reported the demonstration of linearly coupled antiregular polariton states, with ordered phase transitions and vortex-pair-petal states at the coalescence threshold, using a non-resonant ring pumping method in a planar perovskite microcavity (Figure 9a). In contrast with the uniform pumping in perovskite microcavities, which creates excitons throughout the microporous plates, toroidal pumping promotes the localised production of polarisable excitons (EPs). These EPs undergo repulsive interactions, leading to the toroidal localised blueshift and potential trap.

Optical communication: optical communication involves the transmission of optical signals from a transmitter to a receiver. Topological lasers are critical to optical communications because they produce highly efficient, high-power beams. The use of topological lasers enables longer transmission distances and higher transmission rates in optical communications. Wang et al [68] observed electromagnetic chiral edge states (CESs) for the first time by using magneto-optical (PhCs) fabricated in the microwave state. Similar to their electronic counterparts, these photonic CESs can only propagate in one direction. The team measured a difference of nearly 50 dB between forward and reverse transmissions, as seen in Figure 9b.

As a new generation of high-brightness surface emitters, TCSEL can be directly extended to any other wavelength range and has a very wide application prospect. It is capable of reaching 10 W peak power in a single device at 1550 nm, the wavelength at which fibre losses are minimal in global communications and the eye-safe wavelength for lidar in self-driving cars. It uses topological cavity technology, has a far-field divergence angle of less than 1° and a side-mode suppression ratio of 60 dB. It can integrate two-dimensional multi-wavelength arrays. The topological properties of TCSEL may mean that larger, more stable single-mode devices can be manufactured with better yield. In addition, it can integrate two-dimensional multi-wavelength arrays, and the topological properties of TCSEL can mean that larger, more stable single-mode devices have better manufacturing yields [11].

Silicon-based photonic circuits are a technology that utilizes silicon-based materials for photonic integration. They are programmable and scalable, making them an ideal platform for realizing photonic circuits. Figure 9c showcases an integrated, tens-of-microwatts, thin-film lithium niobate photonic circuit [69]. The circuit uses two input paths to drive resonant cavities. These cavities exhibit fundamental and second harmonic frequencies, shown in red and blue, respectively. The researchers used tunable lasers operating at communication wavelengths to study second harmonic generation and cascade parametric oscillation processes through their optical paths. One of the input paths is connected to a shorter wavelength laser and is used to drive a direct optical parametric oscillator (OPO). Light is coupled in and out of the chip using a lensed fibre. The output light is separated and sent to silicon and InGaAs avalanche photodiodes [19], a free-space setup is used that has a dichroic reflector.

**Figure 10:**
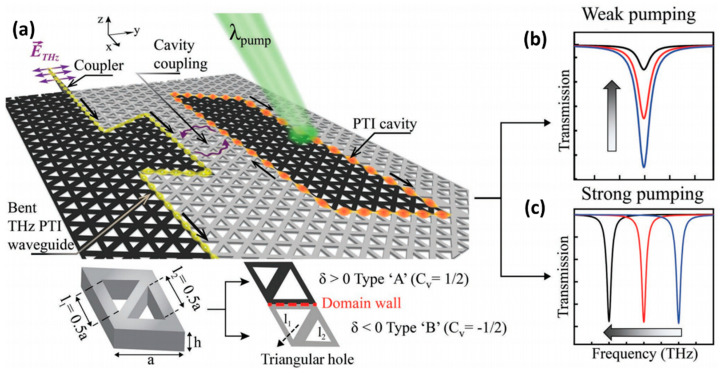
All-optical control of a THz topological cavity-waveguide chip, reproduced with permission from [70], copyright 2022, *Advanced Materials*: (**a**) Artistic illustration of a valley photonic crystal (VPC) cavity-waveguide chip on an all-silicon (Si) platform. A continuous laser with wavelength 532 nm (energy 2.33 eV) photoexcites the Si above the bandgap (1.1 eV). The inset shows the unit cell of the VPC, where a denotes the periodicity and h represents the height equal to 200 μm. The unit cell contains two triangular air holes of side lengths *I*_1_ and *I*_2_. δ = *I*_2_ − *I*_1_ denotes the degree of asymmetry, with δ > 0 and δ < 0 corresponding to Type A and Type B VPCs (shaded in black and grey in the schematic). (**b**) Schematic illustration of THz intensity modulation caused by photoexciting the domain wall of VPC cavity (The black, red and blue lines represent the transmission intensity when δ > 0, δ < 0 and δ = 0, respectively). (**c**) Schematic representation of frequency agility of the topological cavity resonance upon high-power photoexcitation.

Terahertz Silicon Interconnection technology is a new communication technology that can realise high-speed, low-power, high-density data transmission in the terahertz frequency band. To meet the growing demand for high-speed, high-capacity consumer electronics products, increased interconnect capacity in terms of bandwidth density and active tunability is required to improve throughput and power efficiency. Low-loss, higher bandwidth terahertz silicon interconnects provide a solution to existing inter-chip/in-chip bandwidth density and power efficiency bottlenecks. Kumar et al. [69] proposed a low-loss terahertz topology interconnection-cavity system that can actively route signals via sharp turns via critical coupling to a topology cavity with an ultra-high quality (Q) factor of 0.2 × 10^6^. The topology cavity consists of a VPC waveguide closed loop (see Figure 10) with a Q of 0.2 × 10^6^, which is the first experimental proof of achieving ultra-high Q resonance at terahertz frequencies. In Figure 10a, the black and grey highlighted regions indicate the topologically distinct VPC domains formed by Type A and Type B unit cells, respectively. It is not difficult to see from Figure 10b,c that the peak frequencies of the two types of transmission intensities under low-power laser excitation are in good agreement.

## 5. Conclusions

This study presents an overview of the research progress made on topological lasers that utilize low-dimensional perovskite materials as the gain medium, and their main applications. Various models of topological lasers have been proposed, with varying materials and structural designs, from a theoretical perspective. Several types of topological lasers exist, such as photonic crystal-based, photonic insulator-based and topological phase-change lasers that incorporate photonic insulators and superconductors. Several research groups have successfully created topological lasers in their experiments, including those based on photonic crystals, photonic insulators with superconductors and phase-change lasers.

In conclusion, the combination of topological laser modes and perovskite materials displays efficient optical emission properties and excellent potential for photonic applications. The structure of topological lasers greatly promotes optical emission from perovskite materials. The perovskite quantum dots exhibit remarkable optical properties and chemical versatility, including a large optical absorption cross-section and exciton binding energy that allows for the efficient absorption of light energy and the generation of excitons. 

In the future, research on the theory and experimental techniques to improve the performance and stability of these dots will focus on combining topological laser modes with Perovskite materials. Therefore, it remains imperative to further investigate the optical properties of low-dimensional perovskite materials and device preparation technologies for enhanced support and the facilitation of topological lasers’ application in diverse fields. Topological lasers serve as an optimal on-chip light source option due to their low threshold, high efficiency, good directionality, high stability, compact size and cost-effectiveness.

## Data Availability

Not applicable.

## References

[1] Wu C., Kumar S., Kan Y., Komisar D., Wang Z., Bozhevolnyi S.I., Ding F. (2022). Room-temperature on-chip orbital angular momentum single-photon sources. Sci. Adv..

[2] Ma R.M., Oulton R.F. (2019). Applications of nanolasers. Nat. Nanotechnol..

[3] Zhong H., Yu Y., Zheng Z., Ding Z., Zhao X., Yang J., Wei Y., Chen Y., Yu S. (2019). Ultra-low threshold continuous-wave quantum dot mini-BIC lasers. Nat. Mater..

[4] Nandi S., Olofsson E., Bertolinom M. (2022). Observation of Rabi dynamics with a short-wavelength free-electron laser. Nature.

[5] Zahidy M., Liu Y., Cozzolino D., Ding Y., Morioka T., Oxenløwe L.K., Bacco D. (2022). Photonic integrated chip enabling orbital angular momentum multiplexing for quantum communication. Nanophotonics.

[6] Liang Y., Li C., Huang Y.Z., Zhang Q. (2020). Plasmonic Nanolasers in On-Chip Light Sources: Prospects and Challenges. ACS Nano.

[7] Huang C.-Y., Zou C., Mao C., Kathryn L.Y., Lee Y.-J., Schlenker C.W., Alex K., Jen Y., Lih Y. (2017). CsPbBr_3_ Perovskite Quantum Dot Vertical Cavity Lasers with Low Threshold and High Stability. ACS Photonics.

[8] Harari G., Bandres M.A., Lumer Y., Rechtsman M.C., Chong Y.D., Khajavikhan M., Christodoulides D.N., Segev M. (2018). Topological insulator laser: Theory. Science.

[9] Shalaev M.I., Walasik W., Tsukernik A., Xu Y., Litchinitser N.M. (2019). Robust topologically protected transport in photonic crystals at telecommunication wavelengths. Nat. Nanotechnol..

[10] Zeng Y., Chattopadhyay U., Zhu B., Qiang B., Li J., Jin Y., Li L., Davies A.G., Linfield E.H., Zhang B. (2020). Electrically pumped topological laser with valley edge modes. Nature.

[11] Yang L., Li G., Gao X., Lu L. (2022). Topological-cavity surface-emitting laser. Nat. Photonics.

[12] Wu J., Ghosh S., Gan Y., Shi Y., Mandal S., Sun H., Zhang B., Liew T.C.H., Su R., Xiong Q. (2023). Higher-order topological polariton corner state lasing. Sci. Adv..

[13] Sheng Y., Liu C., Yu L., Yang Y., Hu F., Sheng C., Di Y., Dong L., Gan Z. (2021). Microsteganography on All Inorganic Perovskite Micro-Platelets By Direct Laser Writing. Nanoscale.

[14] Kim H.R., Hwang M.S., Smirnova D., Jeong K.-Y., Kivshar Y., Park H.-G. (2020). Multipolar lasing modes from topological corner states. Nat. Commun..

[15] Zhu T., Guo C., Huang J., Wang H., Orenstein M., Ruan Z., Fan S. (2021). Topological Optical Differentiator. Nat. Commun..

[16] Ikhioya I.L., Aisida S.O., Ahmad I., Ezema F.I. (2023). The effect of molybdenum dopant on rare earth metal chalcogenide material. Chem. Phys. Impact.

[17] Tian J., Tan Q.Y., Wang Y., Yang Y., Yuan G., Adamo G., Soci C. (2023). Perovskite quantum dot one-dimensional topological laser. Nat. Commun..

[18] Lu L., Joannopoulos J.D., Soljačić M. (2014). Topological photonics. Nat. Photonics.

[19] Li Z., Luo X.-W., Gu Q. (2023). Topological on-chip lasers. APL Photonics.

[20] Nakata K., Kim S.K., Takayoshi S. (2019). Laser Control of Magnonic Topological Phases in Antiferromagnets. Phys. Rev. B.

[21] Pal V., Tradonsky C., Chriki R., Friesem A.A., Davidson N. (2017). Observing Dissipative Topological Defects with Coupled Lasers. Phys. Rev. Lett..

[22] Iwahashi S., Kurosaka Y., Sakai K., Kitamura K., Takayama N., Noda S. (2011). Higher-order vector beams produced by photonic-crystal lasers. Opt. Express.

[23] Lisa S. (2019). Back to Basics: Laser Safety. AORN J..

[24] Bahari B., Ndao A., Vallini F., El Amili A., Fainman Y., Kanté B. (2017). Nonreciprocal lasing in topological cavities of arbitrary geometries. Science.

[25] Yang Z.-Q., Shao Z.-K., Chen H.-Z., Mao X.-R., Ma R.-M. (2020). Spin-Momentum-Locked Edge Mode for Topological Vortex Lasing. Phys. Rev. Lett..

[26] Smirnova D., Tripathi A., Kruk S., Hwang M.-S., Kim H.-R., Park H.-G., Kivshar Y. (2020). Room-temperature lasing from nanophotonic topological cavities. Light-Sci. Appl..

[27] St-Jean P., Goblot V., Galopin E., Lemaitre A., Ozawa T., Gratiet L.L., Sagnes I., Bloch J., Amo A. (2021). Author Correction: Lasing in topological edge states of a one-dimensional lattice. Nat. Photonics.

[28] Jae-Hyuck C., William E.H., Yuzhou G.N.L., Midya P., Babak B., Demetrios N.C., Mercedeh K. (2021). Room temperature electrically pumped topological insulator lasers. Nat. Commun..

[29] Dierks H., Zhang Z., Lamers N., Wallentin J. (2023). 3D X-ray microscopy with a CsPbBr_3_ nanowire scintillator. Nano Res..

[30] Volpe L., Fedosejevs R., Gatti G., Pérez-Hernández J.A., Méndez C., Apiñaniz J., Vaisseau X., Salgado C., Huault M., Malko S. (2019). Generation of high energy laser-driven electron and proton sources with the 200 TW system VEGA 2 at the Centro de Laseres Pulsados. High Power Laser Sci. Eng..

[31] Deng J., Dong H., Zhang C., Wu Y., Yuan J., Zhu X., Jin F., Li H., Wang Z., Cai H. (2022). Observing the quantum topology of light. Science.

[32] Ivan A., Iacopo C. (2020). Theory of The Coherence of Topological Lasers. Phys. Rev. X.

[33] Noginov M.A., Zhu G., Belgrave A.M., Bakker R., Shalaev V.M., Narimanov E.E., Stout S., Herz E., Suteewong T., Wiesner U. (2009). Demonstration of a spaser-based nanolaser. Nature.

[34] Ota Y., Katsumi R., Watanabe K., Iwamoto S., Arakawa Y. (2018). Topological photonic crystal nanocavity laser. Commun. Phys..

[35] Henriques G.C.J., Rappoport G.T., Bludov V.Y., Vasilevskiy M.I., Pereset N.M.R. (2020). Topological photonic Tamm states and the Su-Schrieffer-Heeger model. Phys. Rev. A.

[36] Wang Z., Chong Y.D., Joannopoulos J.D., Soljacić M. (2008). Reflection-Free One-Way Edge Modes in A Gyromagnetic Photonic Crystal. Phys. Rev. Lett..

[37] Iserles A., Kropielnicka K., Singh P. (2019). Compact schemes for laser-matter interaction in Schrödinger equation based on effective splittings of Magnus expansion. Comput. Phys. Commun..

[38] Lu Y.-J., Kim J., Chen H.-Y., Wu C., Dabidian N., Sanders C.E., Wang C.-Y., Lu M.-Y., Li B.H., Qiu X. (2012). Plasmonic Nanolaser Using Epitaxially Grown Silver Film. Science.

[39] Li Y.J., Lv Y., Zou C.-L., Zhang W., Yao J., Zhao Y.S. (2016). Output Coupling of Perovskite Lasers from Embedded Nanoscale Plasmonic Waveguides. J. Am. Chem. Soc..

[40] Zhu W., Xu T., Wang H., Zhang C., Deotare P.B., Agrawal A., Lezec H.J. (2017). Surface plasmon polariton laser based on a metallic trench Fabry-Perot resonator. Sci. Adv..

[41] Fernandez-Bravo A., Wang D., Barnard E.S., Teitelboim A., Tajon C., Guan J., Schatz G.C., Cohen B.E., Chan E.M., Schuck P.J. (2019). Ultralow-threshold, continuous-wave upconverting lasing from subwavelength plasmons. Nat. Mater..

[42] Han C., Lee M., Callard S., Seassal C., Jeon H. (2019). Lasing at topological edge states in a photonic crystal L3 nanocavity dimer array. Light Sci. Appl..

[43] Li H., Zachariah A., Eugene J.M., Zhen B. (2020). Quadrupole Topological Photonic Crystals. Nat. Commun..

[44] Mukherjee S., Rechtsman M.C. (2021). Observation of Unidirectional Solitonlike Edge States in Nonlinear Floquet Topological Insulators. Phys. Rev. X.

[45] Hasan M.Z., Kane C.L. (2010). Colloquium: Topological Insulators. Rev. Mod. Phys..

[46] von Klitzing K. (2005). Developments in the quantum Hall effect. Philos. Trans. R. Soc. A.

[47] Lan Z., Chen M.L., Gao F., Zhang S., Wei E.I. (2022). A brief review of topological photonics in one, two, and three dimensions. Rev. Phys..

[48] Ozawa T., Price M.H., Amo A., Goldman N., Hafezi M., Lu L., Rechtsman M.C., Schuster D., Simon J., Zilberberg O. (2019). Topological photonics. Rev. Mod. Phys..

[49] Han B., Rao Y., Wu H., Yao J., Guan H., Ma R., Wang Z. (2020). Low-noise high-order Raman fiber laser pumped by random lasing. Opt. Lett..

[50] Shukai M., Steven M.A. (2020). Microwave Applications of Photonic Topological Insulators. Appl. Phys. Lett..

[51] Tokura Y., Yasuda K., Tsukazaki A. (2019). Magnetic Topological Insulators. Nat. Rev. Phys..

[52] Huang S., Kim K., Efimkin D.K., Lovorn T., Taniguchi T., Watanabe K., MacDonald A., Tutuc Emanuel H., LeRoy B.J. (2019). Topologically Protected Helical States in Minimally Twisted Bilayer Graphene. Phys. Rev. Lett..

[53] Pan Y., Wang L., Zhang Y., Su X., Gao D., Chen R., Huang L., Sun W., Zhao Y., Gao D. (2022). Multi-Wavelength Laser Emission by Hot-Carriers Transfers in Perovskite-Graphene-Chalcogenide Quantum Dots. Adv. Opt. Mater..

[54] Han S., Chua Y., Zeng Y., Zhu B., Wang C., Qiang B., Jin Y., Wang Q., Li L., Giles A. (2023). Photonic Majorana quantum cascade laser with polarization-winding emission. Nat. Commun..

[55] Hafezi M., Taylor J.M. (2014). Topological physics with light. Phys. Today.

[56] Chen Y., Feng J., Huang Y., Chen W., Su R., Ghosh S., Hou L., Xiong  Q., Qiu C.W. (2023). Compact spin-valley-locked perovskite emission. Nat. Mater..

[57] Pan Y., Wang L., Su X., Gao D., Chen R., Zhang Y., Zhao Y., Li L., Gao D. (2022). The effectively optical emission modulation in perovskite MAPbBr(3) crystal by hot-electron transfer from metals. J. Phys. D Appl. Phys..

[58] Pu J., Zhang W., Matsuoka H., Kobayashi Y., Takaguchi Y., Miyata Y., Matsuda K., Miyauchi Y., Takenobu T. (2021). Room-Temperature Chiral Light-Emitting Diode Based on Strained Monolayer Semiconductors. Adv. Mater..

[59] Lin Y.Y., Li Y.W. (2022). Spiral-Phase-Defect Resonator and Its Application in Vortex Laser of Controllable Topological Charges. IEEE Photonics J..

[60] Liao K., Zhong Y., Du Z., Liu G., Li C., Wu X., Deng C., Lu C., Wang X., Chan C.T. (2023). On-chip integrated exceptional surface microlaser. Sci. Adv..

[61] Urban S., Andres F., Tudor O., Micheletti P., Cibella S., Torrioli G., Beck M., Faist J., Scalari G. (2022). Planarized THz quantum cascade lasers for broadband coherent photonics. Light Sci. Appl..

[62] Zhou C., Qi Y., Zhang S., Niu W., Wu S., Ma W., Tang B. (2022). Water rewriteable double-inverse opal photonic crystal films with ultrafast response time and robust writing capability. Chem. Eng. J..

[63] Zhang W., Xie X., Hao H., Dang J., Xiao S., Shi S., Ni H., Niu Z., Wang C., Jin K. (2020). Low-threshold topological nanolasers based on the second-order corner state. Light Sci. Appl..

[64] Zhitong L., Luo X.-W., Lin D., Gharajeh A., Moon J., Hou J., Zhang C., Gu Q. (2023). Topological Microlaser with A non-Hermitian Topological Bulk. Phys. Rev. Lett..

[65] Wang Y., Li X., Nalla V., Zeng H., Sun H. (2017). Solution-Processed Low Threshold Vertical Cavity Surface Emitting Lasers from All-Inorganic Perovskite Nanocrystals. Adv. Funct. Mater..

[66] Haldane F.D.M., Raghu S. (2008). Possible Realization of Directional Optical Waveguides in Photonic Crystals with Broken Time-Reversal Symmetry. Phys. Rev. Lett..

[67] Zhang S., Zhu Z., Du W., Wu X., Ghosh S., Zhang Q., Xiong Q., Liu X. (2023). All-Optical Control of Rotational Exciton Polaritons Condensate in Perovskite Microcavities. ACS Photonics.

[68] Wang Z., Chong Y., Joannopoulos J.D., Soljacic M. (2009). Observation of Unidirectional Backscattering-immune Topological Electromagnetic States. Nature.

[69] McKenna T.P., Stokowski H.S., Ansari V., Mishra J., Jankowski M., Sarabalis C.J., Herrmann J.F., Langrock C., Fejer M., Amir M. (2022). Ultra-low-power second-order nonlinear optics on a chip. Nat Commun..

[70] Kumar A., Gupta M., Pitchappa P., Tan T.C., Chattopadhyay U., Ducournau G., Wang N., Chong Y., Singh R. (2022). Active Ultrahigh-Q (0.2 × 10^6^) THz Topological Cavities on a Chip. Adv. Mater..

